# A Method of Realizing Adaptive Uniform Illumination by Pyramid Prism for PA-LiDAR

**DOI:** 10.3390/mi15101232

**Published:** 2024-09-30

**Authors:** Shuo Zhang, Baiyang Wu, Yong Bi, Weinan Gao

**Affiliations:** 1 Applied Laser Research Center, Technical Institute of Physics and Chemistry, Chinese Academy of Sciences, Beijing 100190, China; zhangshuo@mail.ipc.ac.cn (S.Z.); wubaiyang22@mails.ucas.ac.cn (B.W.); wngao@mail.ipc.ac.cn (W.G.); 2 University of Chinese Academy of Sciences, Beijing 100049, China

**Keywords:** laser radar, radar imaging, beam shaping, illumination

## Abstract

In this paper, we propose a simple method to generate the uniform illumination using a pyramid prism for Plane Array Laser Imaging Detection and Ranging (PA-LiDAR). The principle of the pyramid prism shaping the Gaussian beam to form a uniform beam was analyzed theoretically. By changing the parameters of the pyramid prism and laser beam, the profile distribution of the output beam can be easily adjusted. Based on the operation mode and illumination requirements of PA-LiDAR, we have developed a set of LiDAR prototypes using a pyramid prism and carried out experimental research on these prototypes. The simulation and experimental results demonstrated that this method can achieve a uniform illumination beam with excellent propagation properties for meeting the technical requirements of PA-LiDAR. This method of uniform illumination has the advantages of being simple, flexible, easily adjustable and convenient to operate.

## 1. Introduction

**Figure 1:**
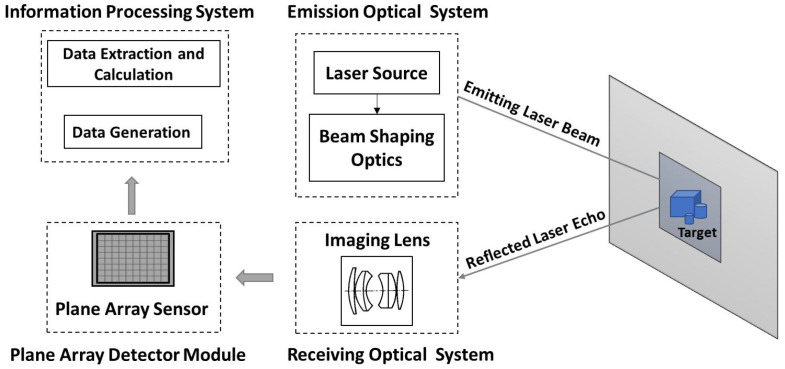
The system components and working principle of PA-LiDAR.

PA-LiDAR, also known as staring imaging laser radar, has emerged as a hot topic in the field of space exploration. Due to its advantages of high measurement accuracy, abundant information, good parallel real-time performance, low power consumption, and small system volume, the PA-LiDAR plays an important role in many fields, such as scene perception, real-time location and map building, scene identification and classification, virtual reality, automatic driving, industrial online detection, and so on [1,2,3,4,5,6]. The emission optical system controls the shaped laser to form an illumination surface covering the whole target scene. The reflected echo is received by the receiving optical system, and each pixel of the plane array sensor obtains the corresponding signal simultaneously. Subsequently, the three-dimensional information of the target is extracted by the information processing system. The working principle of PA-LiDAR is shown in Figure 1.

In the detection process of PA-LiDAR, the measurement accuracy and imaging quality are significantly affected by illumination, so it is very important to obtain a uniform and high-energy-efficiency illumination beam. The illumination system of PA-LiDAR is composed of laser sources and illumination-shaping optic elements. Semiconductor lasers, or laser diodes, possess the characteristics of high output power, high photoelectric conversion efficiency, convenient laser intensity modulation, compact size and long lifetime, making them highly suitable as active illumination sources for PA-LiDAR [7,8,9,10]. Due to their waveguide cavity structure, the semiconductor laser output works on multimode, which makes the spatial distribution of the beam asymmetrical, and the far-field light intensity generally presents a near-Gaussian distribution. However, such beam characteristics cannot meet the illumination requirements of PA- LiDAR. Because the asymmetry of the illumination field of view does not match the receiving surface of the detection sensor, it is not conducive to making full use of energy, and the uneven distribution of intensity in the illumination field of view leads to a reduction in the available dynamic range of the receiving system. Therefore, the requirements for the illumination optical system of PA-LiDAR should meet as follows: (1) Field of view matching (2) High-quality uniformity (3) Good distance transmission characteristics, that is, large dynamic range. To meet these requirements, beam-shaping techniques suitable for application in PA-LiDAR must be studied.

Various kinds of methods for beam shaping to produce uniform illumination have been proposed, such as diffusers [11], DOE (diffraction optical elements), binary diffraction gratings [12], LC-SLM (liquid crystal spatial light modulators) [13], fly-eye lens [14,15], fibers [16,17], and integrator rods [18]. Table 1 describes the evaluation of these beam-shaping methods.

These beam-shaping methods are not suitable for or meet the illumination requirements of PA-LiDAR due to their various shortcomings [19,20,21,22,23,24,25]. In this paper, we proposed a simple method to obtain the uniform illumination for PA-LiDAR via pyramid prism. The pyramid prism itself has the characteristics of splitting beam. The incident beam is divided by the pyramid prism and then superimposed on the target surface to form a flat-top beam for illumination. The influence of optical and structural parameters, such as the incidence angle of laser sources and the side length, included angle, height of the pyramid prism, were simulated and analyzed. According to the optimal structure parameters of simulation calculation, the corresponding optical components are fabricated and verified in the PA-LiDAR prototype. Simulation and experimental results have shown that the rectangular uniform illumination beam can be achieved by this method, which has the advantages of adjustable profile distribution and good transmission characteristics. The structure of this technological method is simple and compact, which is helpful to reduce the size and complexity of shaping device. It is very suitable for solid-state laser radar illumination system.

## 2. Description of Beam-Shaping Progress

### 2.1. Basic Principles of Operation

**Figure 2:**
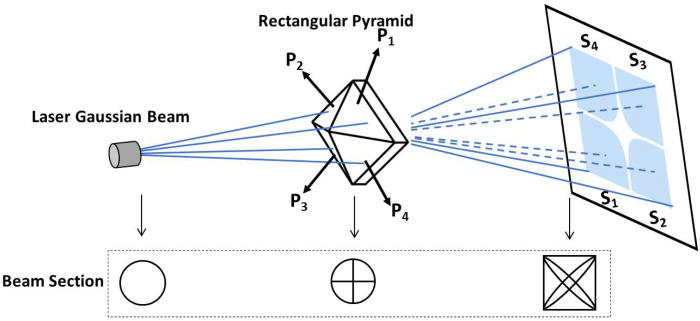
Principle of beam shaping progress by rectangular pyramid.

The generation of a shaped flat top beam using a rectangular pyramid is shown in Figure 2. The laser beam with a Gaussian intensity profile passes through a pyramid prism. The pyramid prism splits the beam on its vertex in four uniform part beams which propagates in opposite directions in pairs to achieve superposition, to form a flat top beam for illumination. It is necessary to take into account the uniformity of flat top and the efficiency of shaping, and select the appropriate value.

**Figure 3:**
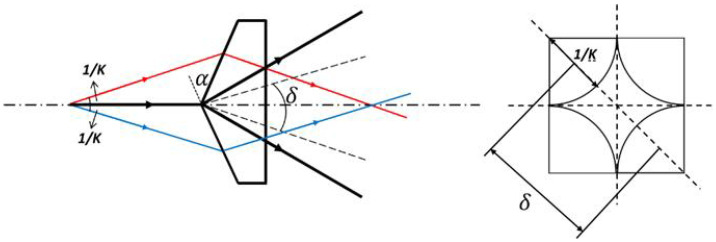
Geometry characteristics of the splitting effects of the pyramid prism.

Referring to Figure 3 [26], the angular separation between the axes of two beams split by two opposed faces of the pyramid prism is given by

(1) $$\delta =\left(n-1\right)\alpha$$

α is the angle between the two faces of pyramid prism, *n* is the refractive index of the pyramid glass, and *K* is the F-number of the divergent beam. To achieve the superposition of the split beams $\delta \leq \sqrt{2}/K$, according to Equation (1),

(2) $$\alpha \leq \frac{\sqrt{2}}{2\left(n-1\right)K}$$

**Figure 4:**
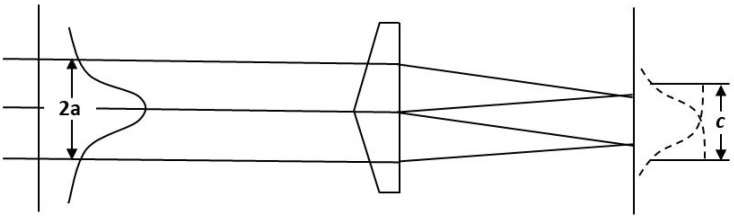
Beam profile shaping with the pyramid prism.

Figure 4 shows the intensity illumination diagram of Gaussian laser beam and flat-top beam with pyramid prism.

**Figure 5:**
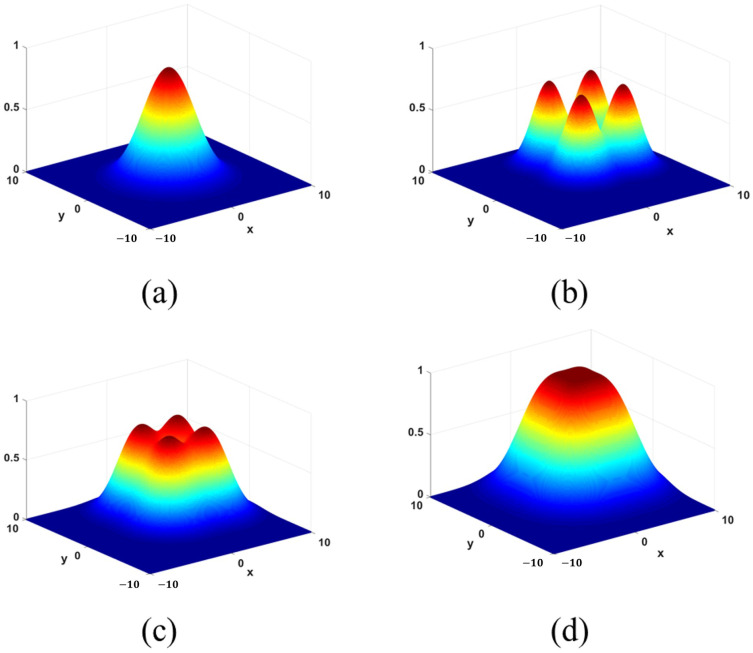
(**a**) Incident Gaussian beam (**b**) Unformed beam (**c**) Unformed beam (**d**) Flat-top beam.

The intensity distribution of the beam illumination obtained with this concept is expressed as the following equations:

(3) $$I\left(x,y\right)=A\left(-2\frac{x^{2}+y^{2}}{a^{2}}\right)+A\left(-2\frac{{\left(x-c\right)}^{2}+y^{2}}{a^{2}}\right)+\cdots +A\left(-2\frac{x^{2}+{\left(y-c\right)}^{2}}{a^{2}}\right)+A\left(-2\frac{{\left(x-c\right)}^{2}+{\left(y-c\right)}^{2}}{a^{2}}\right)$$

where *a* is the spot size of Gaussian beam and *c* is the side length of the square region of flat-top beam. Based on Equation (2) and discussion above, *c* is determined by the parameters of incident Gaussian beam and pyramid prism, and flat top beam can be formed only when certain relationship is satisfied, as shown in Figure 5.

### 2.2. Numerical Simulation and Analysis

**Figure 6:**
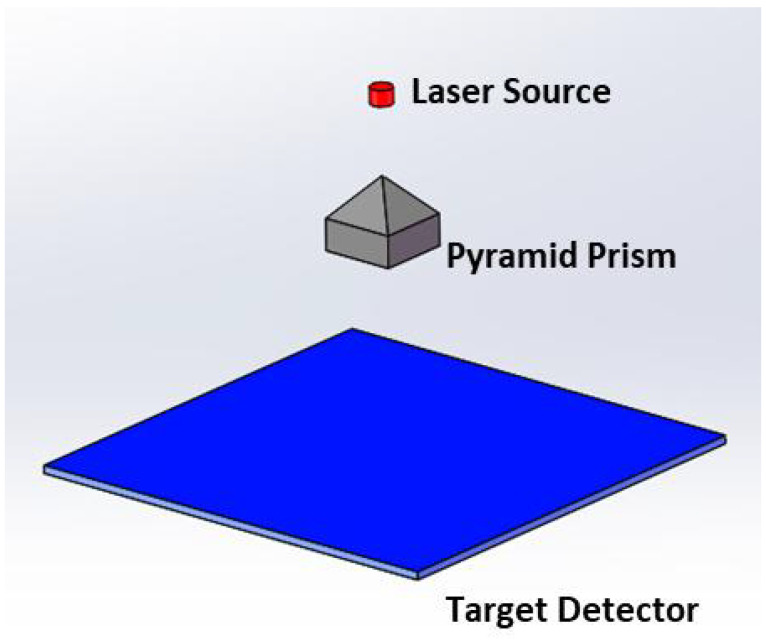
Simulation model.

A solid model of the illumination system was established in software platform. The model is shown in Figure 6.

**Figure 7:**
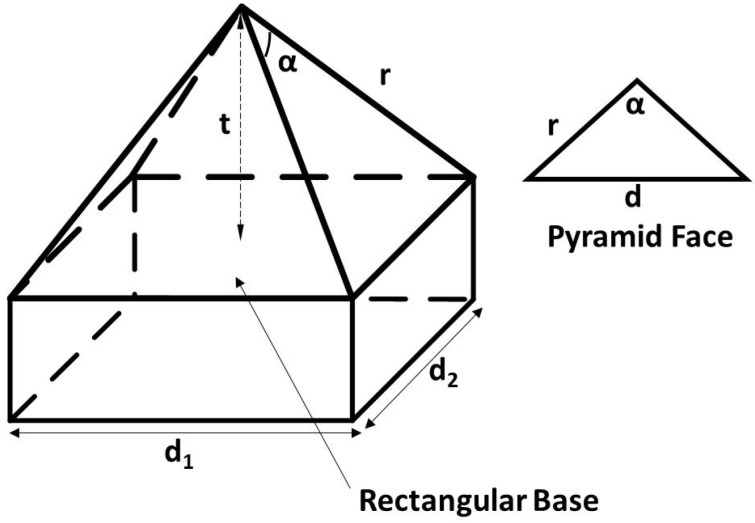
Geometric structure of rectangular pyramid.

To facilitate the simulation analysis, several parameters of the pyramid prism are defined as shown in Figure 7. The dependence of side length *d* of rectangular base, edge length *r*, included angle α and height *t* of pyramid face and transverse field intensity distribution of the flat-top beam on the included angle α of the pyramid prism and the incidence angle of the laser beam are analyzed theoretically by simulation.

#### 2.2.1. Included Angle

**Figure 8:**
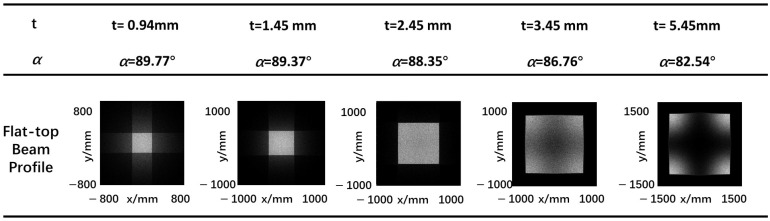
Flat-top laser beam profile at z = 50 mm plane produced by different included angles.

From Figure 7, the relationship between the included angle and other parameters can be obtained. Under certain conditions, the included angle α, is uniquely determined by the height *t* of pyramid face. Figure 8 shows the characteristics of a flat-top laser beam at 50 mm transmission distance under several different included angles (different height of pyramid face). The incidence angle of the laser source is set to 15°, and the side length *d* of rectangular base is set to 20 mm.

It can be seen from Figure 8 that with fixed laser source parameters, when included angle α of pyramid face is within a certain range, the rectangular beam with good profile can be formed according to the previous section. Therefore, the desired flat-top laser beam can be obtained by adjusting the included angle or the height of pyramid face.

The main parameters are side length *d* of rectangular base and height *t* of pyramid face, which will cause changes in the profile and size of the output beam. Under certain conditions, the included angle, α, is uniquely determined by the height *t* of pyramid face. When the laser source’s divergence angle is fixed, a larger rectangular side length *d* facilitates the formation of a more uniformly distributed rectangular output light spot, leading to a better result. When the divergence angle of the laser source and the side length *d* are fixed, increasing the pyramid height *t* facilitates the formation of a more uniformly distributed rectangular output beam spot shape, leading to greater practicality.

#### 2.2.2. Incidence Angle of Laser Source

**Figure 9:**
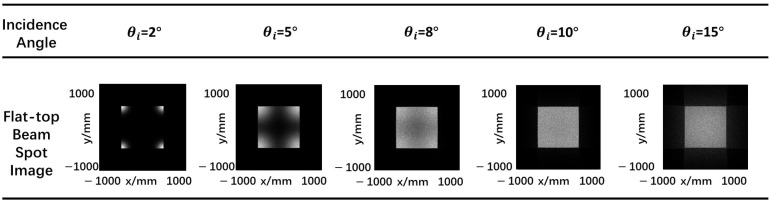
Flat-top laser beam profile at z = 50 mm plane produced by different incidence angles.

According to previous section, the change in the incidence angle of the laser source will result in a change in the profile and size of the output beam. Figure 9 shows the beam profile of the flat-top laser beams for three chosen incidence angles: 2°, 5°, 8°, 10° and 15° at 50 mm transmission distance. The height *t* of pyramid face is set to 1.45 mm and included angle α of pyramid face is set to 89°.

From the simulation results, it can be observed that for the same rectangular pyramid angle, changes in the incident angle of the light source lead to changes in the shape of the output beam spot. Within a certain range, when the incident angle is small, the light spot tends to concentrate at the four corners. As the incidence angle increases, the beam spot gradually forms a more rectangular shape (the angle should not be too large, otherwise the light will exit from outside the edges of the prism).

#### 2.2.3. Propagation Characterization

**Figure 10:**
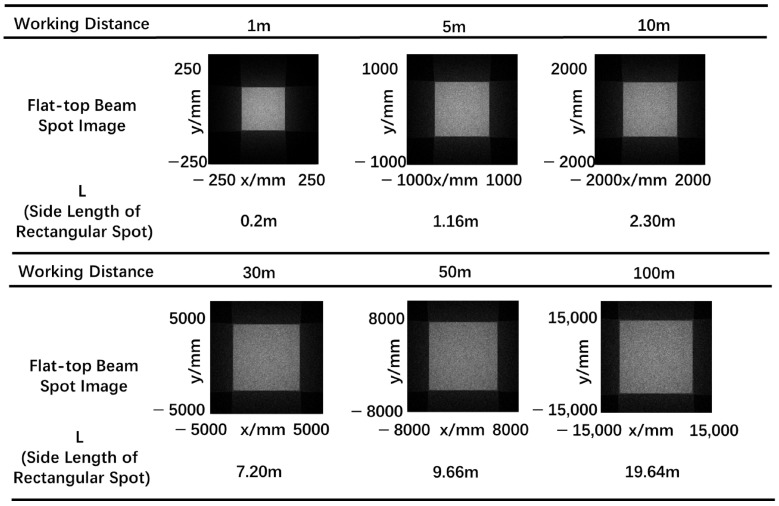
The flat-top laser beam profile with different working distance.

**Figure 11:**
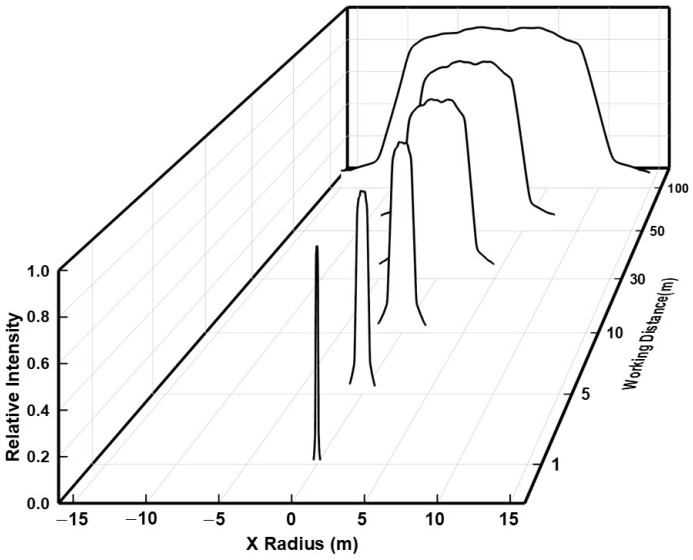
Intensity distribution of the flat-top laser beam with different working distance.

The relationship between the spot image, intensity distribution and working distance is shown in Figure 10 and Figure 11. With the increase of transmission distance, the profile and the uniformity of the output beam keep in good condition. Hence, the output flat-top laser beam has good beam quality and good transmission characteristics.

## 3. PA-LiDAR Prototype Experiment

### 3.1. PA-LiDAR System

**Figure 12:**
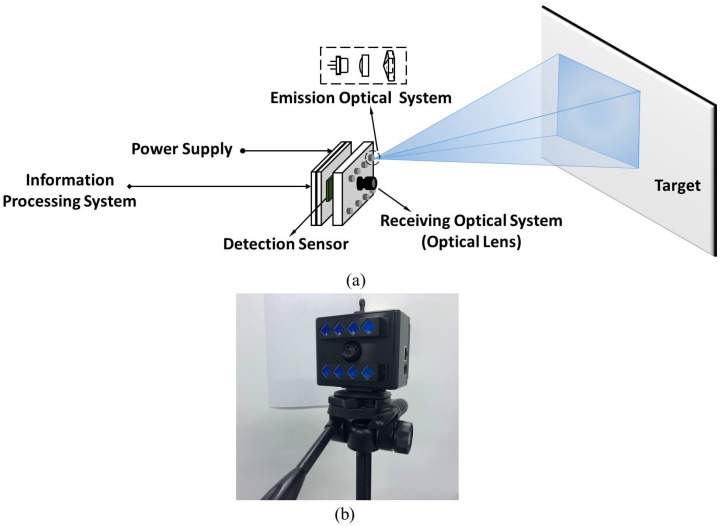
(**a**) Plane array imaging laser radar system structure (**b**) The prototype of PA-LiDAR.

In order to verify and evaluate the practicability, we have applied this method to the PA-LiDAR prototype. It can be seen from Figure 12a, it consists of emission optical system, receiving optical lens (optical lens), detection sensor, information processing system and power supply. The transmitting optical system is composed of groups of laser diode, aspheric lens and rectangular pyramid. The surface figure tolerance of the rectangular pyramid we fabricated is λ/4 at 632.8 nm, the length tolerance is ±0.1 mm, and the angular tolerance is ±3 arcminutes. These groups form an array arrangement and are coaxial with the receiving optical lens. According to the design results of the structure parameters discussed in previous Section, as shown in Figure 12b, eight groups LD (Laser diode) array of 1 W were used to construct the illumination system of prototype.

**Figure 13:**
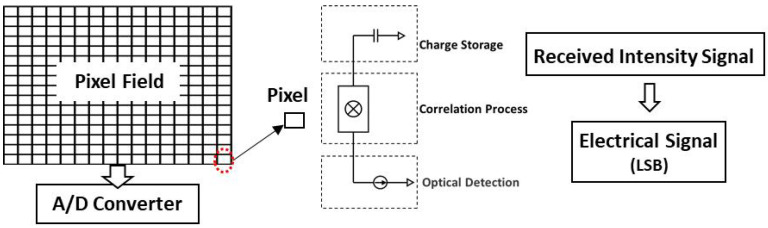
Construction and working mode of detection sensor.

The detection sensor of the PA-LiDAR is a kind of matrix depth sensor, which integrates a great deal of sensor pixels on the array plane. Each pixel of the PA-LiDAR detector demodulates the received optical signal independently, so it can not only measure the time signal but also the amplitude signal. As is shown in Figure 13, when the external light illuminates the pixel array, the photoelectric effect occurs and the corresponding charge is generated in the pixel unit. The photodiode in each pixel converts the light intensity on the surface of its array into electrical signal. The pixel are transmitted to the analog signal processing unit and A/D converter, then reads the electrical signal out. The unit representing the read electrical signal is LSB (Least Significant Bit). The intensity of the target received by the detector is affected by the emission beam energy of the laser source, space transmission loss and target reflectivity. Therefore, for the same scene, we can use the reflectance intensity or amplitude information of the modulated beam to indirectly evaluate and analyze the illumination quality.

### 3.2. Experiment and Analysis of Illumination Uniformity

On the basis of the illumination requirements of PA-LiDAR as mentioned in Section 1 and the related parameters and concept in previous Section, a comprehensive evaluation function of illumination uniformity quality is needed.

**Figure 14:**
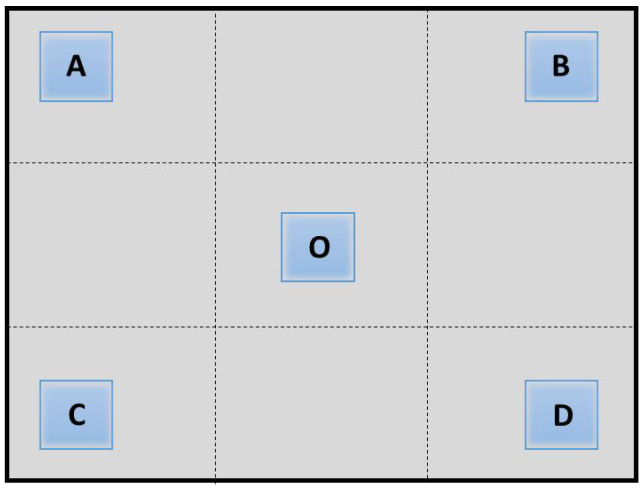
Evaluation function of uniformity calculation. O: 20 × 20 pixel area at the center of the field of view. A,B,C,D: 20 × 20 pixel areas at the four corners of the field of view.

In order to calculate the illumination uniformity, the laser radar were set to measure the blank plane target that fills the entire detection field of view. At the same distance, the reflectivity is consistent in the detection field of view, and the distribution of illumination directly reflects the received energy of the sensor. The following description refers to Figure 14. The average amplitude value $E_{O}$ of 20 × 20 pixels in the central field of view *O* and the average amplitude values $E_{A}$, $E_{B}$, $E_{C}$ and $E_{D}$ of four regions *A*, *B*, *C*, *D* of 90% field of view are calculated, respectively. The uniformity was characterized by the ratio of the average amplitude of the four regions to the average of the central amplitude. The calculation formula is as follows:

(4) $$E_{i}=\frac{E_{A}+E_{B}+E_{C}+E_{D}}{4}$$

(5) $$K=\frac{E_{i}}{E_{O}}$$

As shown by the data provided in Table 2, in several different detection distance, the same target scene was detected by flat-top beam shaped by rectangular pyramid and non-shaped Gaussian beam, respectively, and the amplitudes was read for calculation. According to Equations (4) and (5), the value of $E_{O}$, $E_{i}$ and *K* were calculated. The *K* value after beam shaping is larger than that without shaping, and it is closer to 1, which indicates that the illumination uniformity after beam shaping is greatly improved and the uniformity in the whole field of view is better.

**Figure 15:**
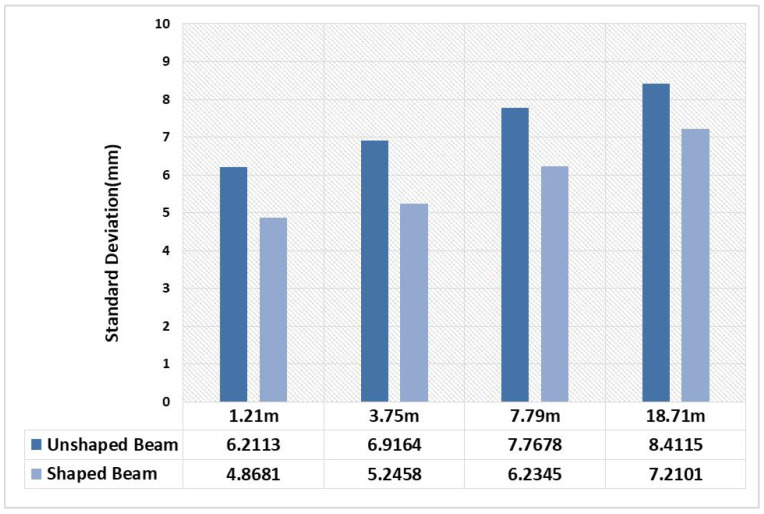
Standard deviation of distance value before and after beam shaping.

Each pixel will produce the system distance measurement error independently. For the plane array imaging laser radar, the ranging accuracy of each point on the whole imaging plane needs to be considered. The relative distance accuracy of each pixel is related to the intensity reflected back to the pixel. In other words, the uniformity of illumination will affect the measurement accuracy and consistency of the whole sensor. In the process of the above experiment, the distance value of several rows of pixel were selected for analysis. Figure 15 shows the standard deviation before and after beam shaping at distance 1.21 m, 3.75 m, 7.79 m, 18.71 m. It can be concluded that the standard deviation of measured distance data after calibration is obviously smaller than that before beam shaping, which shows that the consistency of measured data through calibration is better.

**Figure 16:**
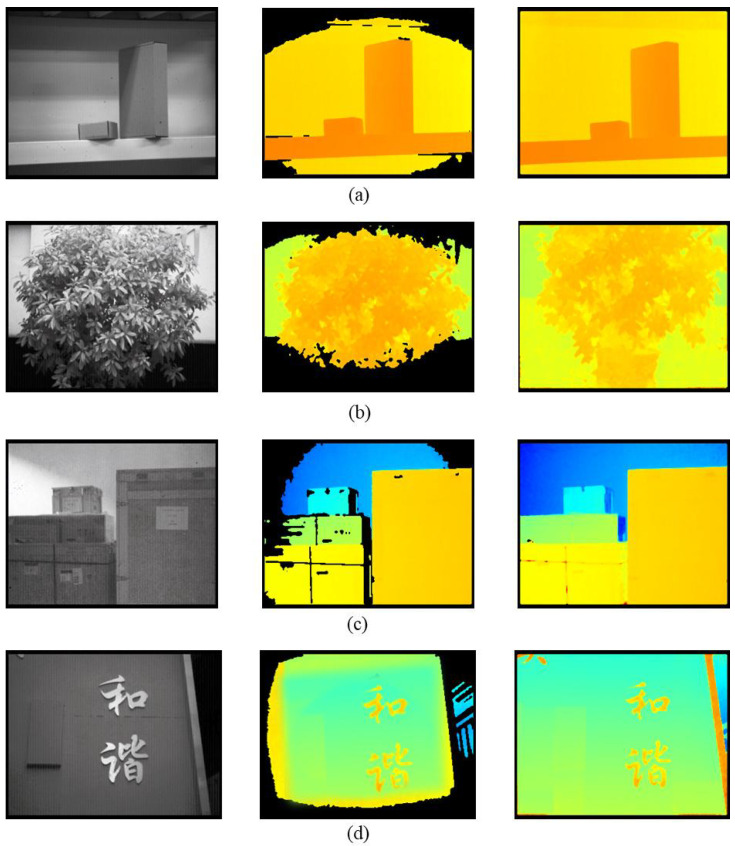
Comparison of the beam before (second column images) and after (fourth column images) shaping in the different actual scenario. (**a**) Objects in laboratory. (**b**) Plants in corridor. (**c**) Goods in warehouse. (**d**) Outdoor signage.

Furthermore, we carried out detection experiments on different scenes with PA-LiDAR prototype. Figure 16a–d shows the comparison before and after beam shaping, including the grayscales and depth Based on the above, one can draw a conclusion that for the same scene, the PA-LiDAR with uniform illumination system after beam shaping can acquire complete and comprehensive image information. We have shown that the uniform illumination with this proposed method is available for the PA-LiDAR in the different distance range and scene.

To summarize, from the above experiment, it can be found that the echo amplitude, the accuracy of measurement and the quality of depth map in real scene are all achieved desired results because of rectangular uniform illumination by this method.

## 4. Conclusions

In this paper, we presented a method obtain uniform illumination for PA-LiDAR using a pyramid prism. The theoretical analysis, simulations, and experimental results showed that the uniform beam produced by this method meets the illumination requirements of PA-LiDAR, exhibiting good adaptability, high quality, and excellent transmission characteristics. This method achieves the desired effect using a single group of optical elements, which helps reduce the size and complexity of the shaping device. Thus, this research provides a novel method for achieving uniform rectangular illumination in solid-state laser radar.

Generally, the design of beam-shaping optical structures can be achieved using only geometric optics tools, and a single pyramid prism set can produce the desired effect. This approach helps to minimize the size and complexity of the shaping device. However, the shape of a pyramid prism is inherently complex, especially when the prism has multiple faces and each face requires precise machining. If only a small quantity of these prisms is produced, the unit price will be relatively high. Batch production is different from single-unit fabrication. Through multiple iterations of processes like wire cutting, grinding, and polishing, combined with strict process control, the fabrication difficulty and cost of pyramid prisms can be significantly reduced.

## Figures and Tables

**Table 1:** Evaluation of Several Typical Beam-Shaping Methods.

Beam Shaping Method	Descriptions
DOE	By means of diffractive optics, the laser beam with Gaussian intensity distribution is shaped into a spot with uniform intensity distribution. The spot shape can be circular, square, rectangular or straight.
	This method can simply realize the flattop illumination, but the illumination pattern could not propagate like a laser beam.
Binary Diffraction Gratings	It has the characteristics of high diffraction efficiency and adjustable spot profile, and can realize the functions of micro, array, integration and arbitrary wavefront.
	The flatness of illumination was insufficient since the beam shape was expressed with the quasi-Sinc function and significantly different from a flattop one.
LC-SLM	A real-time, adjustable method can be used to obtain the near-field beam with desired shape (such as square, circular) with flat top intensity distribution.
	The laser damage threshold is low and the components are expensive.
Fly-eye lens	The fly-eye lens subdivides and expands the non-uniform beam to the entire field of view and superimposes to obtain a uniform illumination area.
	This kind of method has not been applied to long-distance active lighting, and the edge has a considerable proportion of non-uniform areas.
Optical Fiber	The semiconductor laser is coupled to a multi-mode optical fiber, and the light mixing effect of multiple reflections of the optical fiber is used to obtain light intensity homogenization and mode symmetry at the fiber output end, and then project it to the illumination area through the optical system.
	The method has high technical difficulty, low coupling efficiency and high coupling cost.
Integrator Rod	Multiple reflections in the integrator rod are used to form the mixed light effect of multiple images overlapping.
	This kind of method has not been applied to the design of long-distance imaging illumination system.

**Table 2:** Requirements for optical butting.

Distance	Non-Shaped Beam			Shaped Beam		
	$E_{i}$ (LSB)	$E_{o}$ (LSB)	*K*	$E_{i}$ (LSB)	$E_{o}$ (LSB)	*K*
1.21 m	629.28	1100.45	0.57	981.32	1011.08	0.91
3.75 m	775.38	1384.49	0.56	1236.15	1357.96	0.92
7.79 m	646.93	1457.89	0.44	1241.82	1420.02	0.89
18.71 m	419.82	760.76	0.54	475.72	508.26	0.95

## Data Availability

The data presented in this study are available on request from the corresponding author.
